# Efficient genome engineering of *Toxoplasma gondii* using the TALEN technique

**DOI:** 10.1186/s13071-019-3378-y

**Published:** 2019-03-15

**Authors:** Hongmei Chen, Yijia Guo, Yushu Qiu, Huanbin Huang, Changqing Lin, Min Liu, Xiaoguang Chen, Peiliang Yang, Kun Wu

**Affiliations:** 1 Department of Pathogen Biology, Guangdong Provincial Key Laboratory of Tropical Disease Research, School of Public Health, Southern Medical University, Guangzhou, 510515 China; 2 Experimental Animal Center, Nanfang Hospital, Southern Medical University, Guangzhou, 510515 China

**Keywords:** TALEN, *Toxoplasma gondii*, Bradyzoite, Tyrosine hydroxylase 2, *In vivo* imaging

## Abstract

**Background:**

Aromatic amino acid hydroxylase 2 (AAH2) is a bradyzoite-specific upregulated protein that may alter host behaviour by altering the host dopaminergic pathway. To better understand the role of the parasite’s AAH2 in host-parasite interactions, we generated an AAH2 fluorescent marker strain of *T. gondii* using the TALEN technique.

**Methods:**

We generated an AAH2 fluorescent marker strain of *T. gondii*, which was designated PRU/AAH2-eGFP, using the TALEN technique. This strain stably expressed pyrimethamine resistance for screening and expressed enhanced green fluorescent protein (eGFP)-tagged AAH2 in the bradyzoite stage. The bradyzoite conversion of PRU/AAH2-eGFP was observed both *in vitro* and *in vivo*. The fluorescence localization of AAH2 in mouse models of chronic infection was observed by a Bruker *in vivo* imaging system.

**Results:**

Transgenic *T. gondii* was successfully generated by the TALEN system. The eGFP-tagged AAH2 could be detected by *in vivo* imaging.

**Conclusions:**

This study verified the feasibility of using TALEN technology for *T. gondii* research and provided an *in vivo* imaging method for *in vivo* research of bradyzoite-stage proteins.

**Electronic supplementary material:**

The online version of this article (10.1186/s13071-019-3378-y) contains supplementary material, which is available to authorized users.

## Background

*Toxoplasma gondii* is an obligate intracellular protozoan parasite and one of the most widespread zoonotic parasites, and it can infect most warm-blooded animals and humans [1, 2]. This species infects up to a third of the world’s population [3, 4] and represents a serious threat to public health. Most healthy adults usually show symptoms of latent infection after infection with *T. gondii*. At this time, the parasites convert to bradyzoites and exist in the form of cysts in the brain and muscle tissue of the host [5], which causes latent infection and may lead to changes in several functions of the host brain and behaviour [6]. As models for human infections, rodents have been studied extensively as intermediate hosts of *T. gondii* because these animals are commonly preyed upon by felids, which are the only definitive hosts of this parasite [7]. According to behavioural studies, mice with chronic *T. gondii* infection exhibit significant changes in reactions, spatial learning, locomotion, memory and the ability to learn new things [6, 8, 9]. These effects are considered to be a result of manipulation by the parasite to increase mouse susceptibility to predation, which leads to successful transmission of the parasite to the feline host [10, 11]. The bradyzoite stage shows a preference for the brain of its intermediate host, which supports the role of *T. gondii* in host manipulation [12].

Studies have shown that *T. gondii* may alter host behaviour by altering the host dopaminergic pathway and increasing dopamine levels in the brain [13–15]. Tyrosine hydroxylase (TH), a member of the aromatic amino acid hydroxylase (AaaH) family, is widespread in insects, mammals and humans and represents the rate-limiting enzyme in the synthesis of dopamine. The genome of *T. gondii* was found to contain two AaaH-coding sequences, namely AAH1 and AAH2, which encode tyrosine hydroxylases with signal peptides [16]. Studies have shown that AaaHs play an important role in the function of the brain, and the genes encoding these enzymes are among the likely candidates for genes associated with schizophrenia [17]. Meanwhile, latent *T. gondii* infection is one of the factors leading to schizophrenia, and serological surveys of *T. gondii* infection have shown that there is a positive correlation between the rate of seropositivity and incidence of schizophrenia [18, 19]. In contrast with AAH1, which is constitutively expressed in tachyzoites and bradyzoites, AAH2 is specifically upregulated in bradyzoite cysts [16], which is the form of the parasite that is present during chronic infection. Due to its bradyzoite-specific upregulation of expression and unique predicted signal peptide, it is particularly important to further characterize the localization and function of AAH2.

Gene function research is closely associated with gene editing technology. For a long time, transgenic *T. gondii* was constructed by transfecting cells with donor DNA containing a long homologous sequence. However, homologous recombination (HR) occurs at a very low frequency; therefore, the screening and isolation of parasites was time consuming. Newly developed custom-designed nucleases, namely zinc finger nucleases (ZFNs), transcription activator-like effector nucleases (TALENs) and the clustered regularly interspaced short palindromic repeat (CRISPR)/CRISPR-associated (Cas) system, have shown high editing efficiency. However, the assembly of functional ZF proteins with the desired DNA binding specificity is laborious and time consuming because it requires an extensive screening process. Moreover, ZF domains exhibit context-dependent binding preference due to crosstalk between adjacent modules when assembled into a larger array [20]. The discovery of transcriptional activator-like effectors (TALEs) from *Xanthomonas* bacteria [21–25] was a breakthrough that simplified the generation of custom TALE DNA-binding domains with programmable specificity [26, 27]. Similar to ZFNs, a pair of TALENs can be designed to induce a targeted double-strand break (DSB) at the desired chromosomal locus, which is repaired by HR when provided with an exogenous donor plasmid containing homologous sequences flanking the cut site. DNA DSBs generated by targeted nucleases dramatically stimulate HR [27, 28], and TALEN technology has been successfully applied in several species. However, the accessibility of this technology in *T. gondii* has not yet been reported. Recently, due to the convenience and high efficiency of multiplex genome editing, CRISPR/CAS9 has proven to be useful for several types of genome modifications in model organisms, including *T. gondii* [29, 30]. However, in contrast with ZFNs and TALENs, the CRISPR/CAS9 system can tolerate small mismatches, insertions and other mutations in the target sequence, which leads to increased off-target effects [31, 32].

Herein, we chose the *T. gondii* PRU strain [33] to construct an AAH2 fluorescent marker strain using the TALEN technique. The tachyzoite-bradyzoite interconversion of transgenic *T. gondii* was triggered *in vitro* by using high-pH medium and *in vivo* by establishing chronically infected mouse models. The fluorescence of eGFP-tagged AAH2 protein in the brain tissue of mice during the latent phase could be observed by an *in vivo* imaging system, allowing determination of the localization of AAH2 in the animal model and confirming the feasibility of TALEN technology for the study of *T. gondii* gene function.

## Results

### Identification of TALENs plasmids and donor plasmid

The TALENs and homology template were designed to target and tag the AAH2 gene with eGFP to generate transgenic *T. gondii*. Recombinant plasmids were verified by PCR and the double-enzyme cleavage method. TALE-L was cloned into pL62, and TALE-R was cloned into pR54. The recombinant plasmids pTALEN-L and pTALEN-R were verified by the double-enzyme cleavage method with BamHI and PstI.

The SAG1 gene promoter was amplified by PCR and inserted into pTALEN-L digested with *Asc*I/*Spe*I. The recombinant plasmid pTALEN-L-SAG1 was digested with *Asc*I/*Spe*I to indicate successful insertion. The GRA2 gene terminator was amplified *via* PCR and inserted into pTALEN-L-SAG1 digested with *Bgl*II/*Not*I. The recombinant plasmid pTALEN-L-SG was digested with *Bgl*II/*Not*I to indicate successful insertion (Additional file 1: Figure S1a, b).

The GRA2 gene terminator sequence was amplified *via* PCR and inserted into pTALEN-R digested with *Apa*I/*Not*I. The recombinant plasmid pTALEN-R-GRA2 was digested with *Bgl*II/*Not*I to indicate successful insertion. The DHFR* and SAG1 gene promoters were amplified by PCR and inserted into pTALEN-R-GRA2 digested with *Asc*I/*Spe*I. The recombinant plasmid pTALEN-R-DSG was digested with *Asc*I/*Spe*I to indicate successful insertion (Additional file 1: Figure S1c, d).

The AAH2 homologous left arm, eGFP gene, DHFR* and AAH2 homologous right arm were amplified *via* PCR from the recombinant plasmid pZEDY. The fragment ZEDY was also amplified and inserted into pUC19 digested with *BamH*I. The donor plasmid pZEDY was digested with BamHI to indicate successful insertion (Additional file 2: Figure S2).

### Verification of transgenic *T. gondii* PRU/AAH2-eGFP

**Fig. 1:**
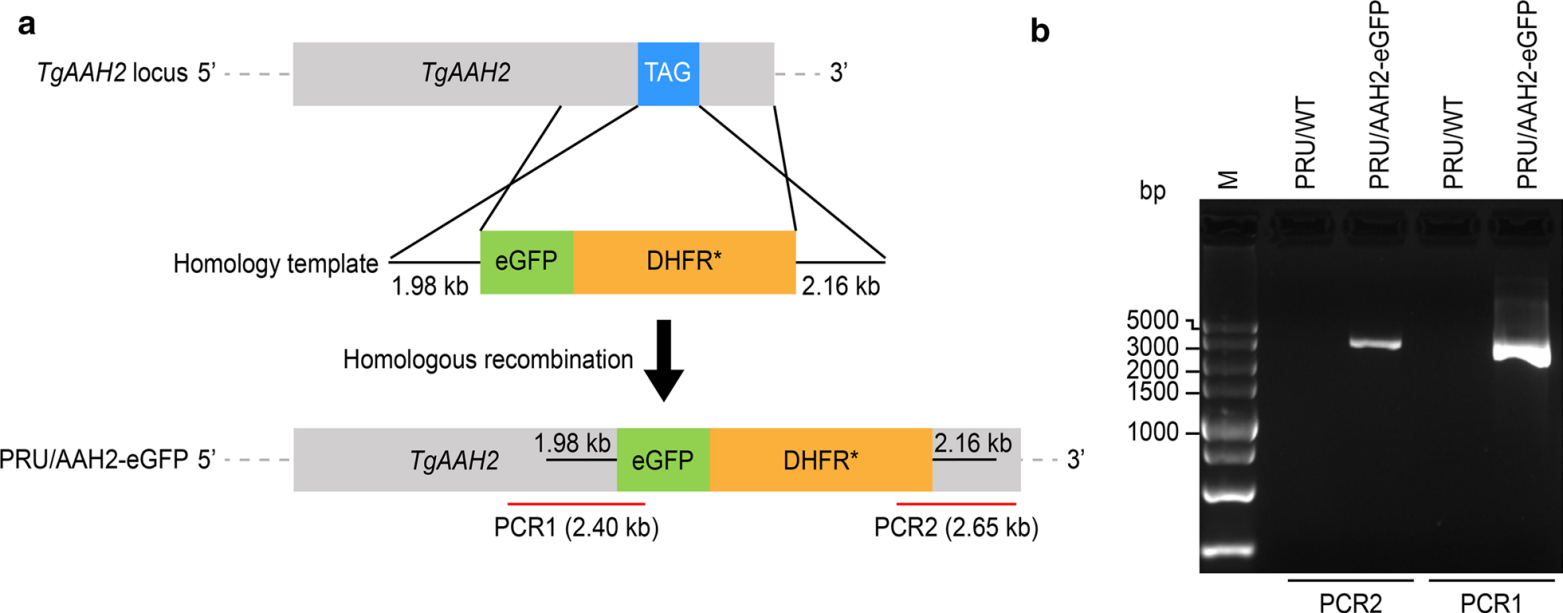
TALEN system tagging of the AAH2 locus. **a** Schematic illustration of the TALEN strategy used to tag AAH2 at the C-terminus with eGFP. **b** Verification PCR showing the correct insertion of eGFP and DHFR*

To further verify the feasibility of the technology used for editing the AAH2 gene, *T. gondii* PRU strain tachyzoites were co-transfected with three recombinant plasmids, namely pTALEN-L-SG, pTALEN-R-DSG and the donor plasmid, and transgenic parasites designed to express fluorescence-tagged AAH2 protein in the bradyzoite stage were identified as follows. The transgenic parasites were screened with pyrimethamine and confirmed by PCR using gDNA as template. The identification PCR primers were designed. PCR1 and PCR2 each had one primer that was not bound on the homologous arm but rather on the toxoplasma genome sequence upstream and downstream of the homologous arms. The fragments amplified by PCR1 and PCR2 both contained part of the inserted genes (eGFP and DHFR*), homologous arms and some genomic sequence beyond the homologous arms (Fig. 1a). A verification PCR assay demonstrated that the eGFP and DHFR* genes were inserted accurately by the TALEN technology (Fig. 1b).

### Expression of AAH2-eGFP in bradyzoites induced *in vitro*

**Fig. 2:**
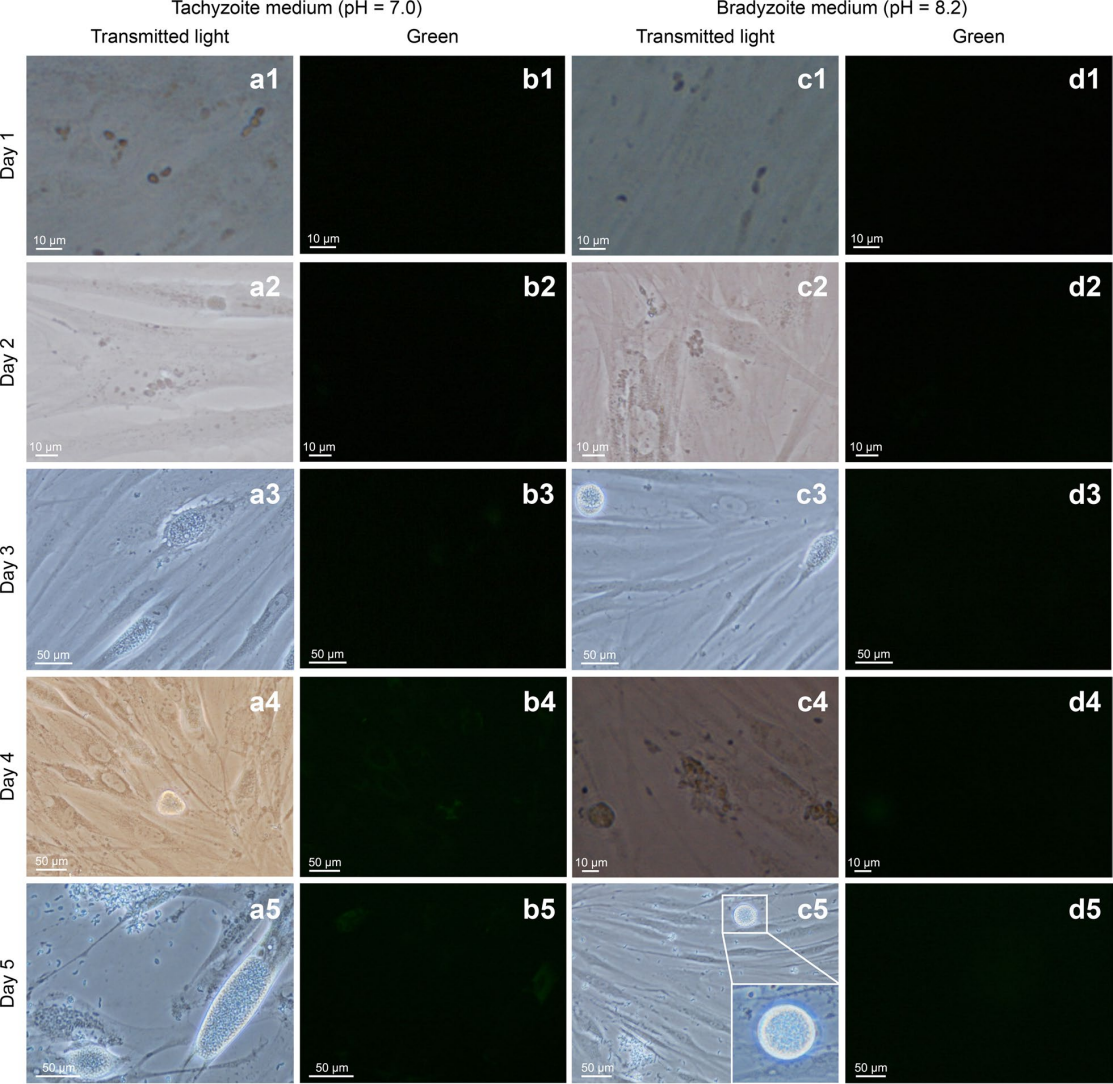
*In vitro* growth of tachyzoite-bradyzoite conversion and fluorescence microscopy analysis of transgenic *T. gondii*. HFF cells were infected with PRU/AAH2-eGFP tachyzoites and cultured in medium at pH 7.0 (**a**, **b**) or pH 8.2 (**c**, **d**). Transmitted light images (**a**, **c**) and green fluorescence images (**b**, **d**) were obtained by fluorescence microscopy. *Scale-bars*: 10 μm, 50 µm

To express the bradyzoite-specific protein AAH2, a tachyzoite-bradyzoite conversion system of transgenic *T. gondii* was established *in vitro*. The status of the parasites and the expression of fluorescence were monitored by inverted fluorescence microscopy. On the first to third days, the parasites invaded the host cell and began to reproduce, and the parasitophorous vacuole could be observed on the third day. The growth and reproduction of the test group was slower than that of the control group (Fig. 2a1-a3, c1-c3), and no specific fluorescence was observed for the first 3 days (Fig. 2b1-b3, d1-d3). On the fourth and fifth days, cyst-like structures could be observed in the test group (Fig. 2c4, c5). Specific fluorescence was not observed in the transgenic bradyzoites induced *in vitro* (Fig. 2b4, b5, d4, d5). However, tachyzoite-bradyzoite conversion *in vitro* cannot fully reflect the expression of bradyzoite-stage proteins *in vivo*.

**Fig. 3:**
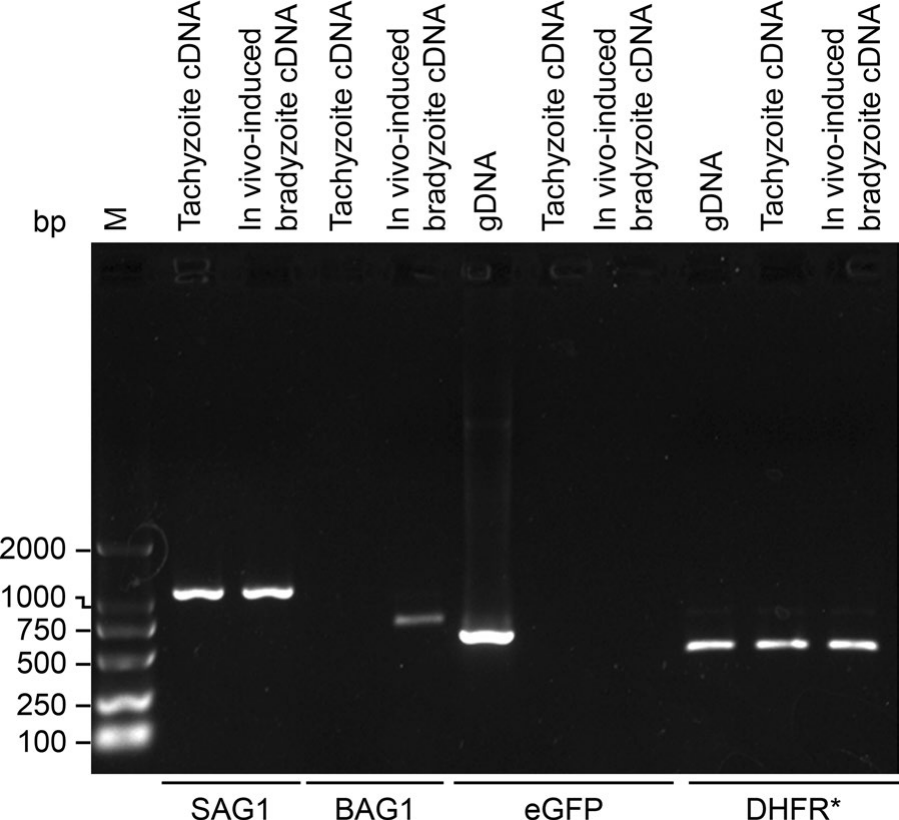
Identification of *in vitro*-induced bradyzoites and expression of inserted genes in transgenic *T. gondii*. RT-PCR verification of the tachyzoite-specific protein SAG1 and bradyzoite-specific protein BAG1 in PRU/AAH2-eGFP tachyzoites and *in vitro*-induced bradyzoites, respectively. RT-PCR verification of the expression of the inserted genes eGFP and DHFR* compared with the PCR verification of the inserted genes from transgenic *T. gondii* gDNA

RNA from the parasites in the control and test groups was extracted to establish a cDNA library for further identification. Bradyzoites induced *in vitro* in the test group were verified by RT-PCR using the bradyzoite-specific BAG1 primers P27 and P28. The tachyzoite-specific SAG1 was amplified from cDNA using the primers P29 and P30 as a control. The verification PCR assay showed that the *in vitro* induction of transgenic *T. gondii* bradyzoites was successful (Fig. 3). The expression of eGFP and DHFR* was identified by RT-PCR using the primers P31/P32 and P33/P34. Verification PCR showed that the resistance gene was expressed at both the tachyzoite stage and the *in vitro*-induced bradyzoite stage, as expected; however, the expression of eGFP was not detected in the test group (Fig. 3).

### Expression of AAH2-eGFP in bradyzoites induced *in vivo*

**Fig. 4:**
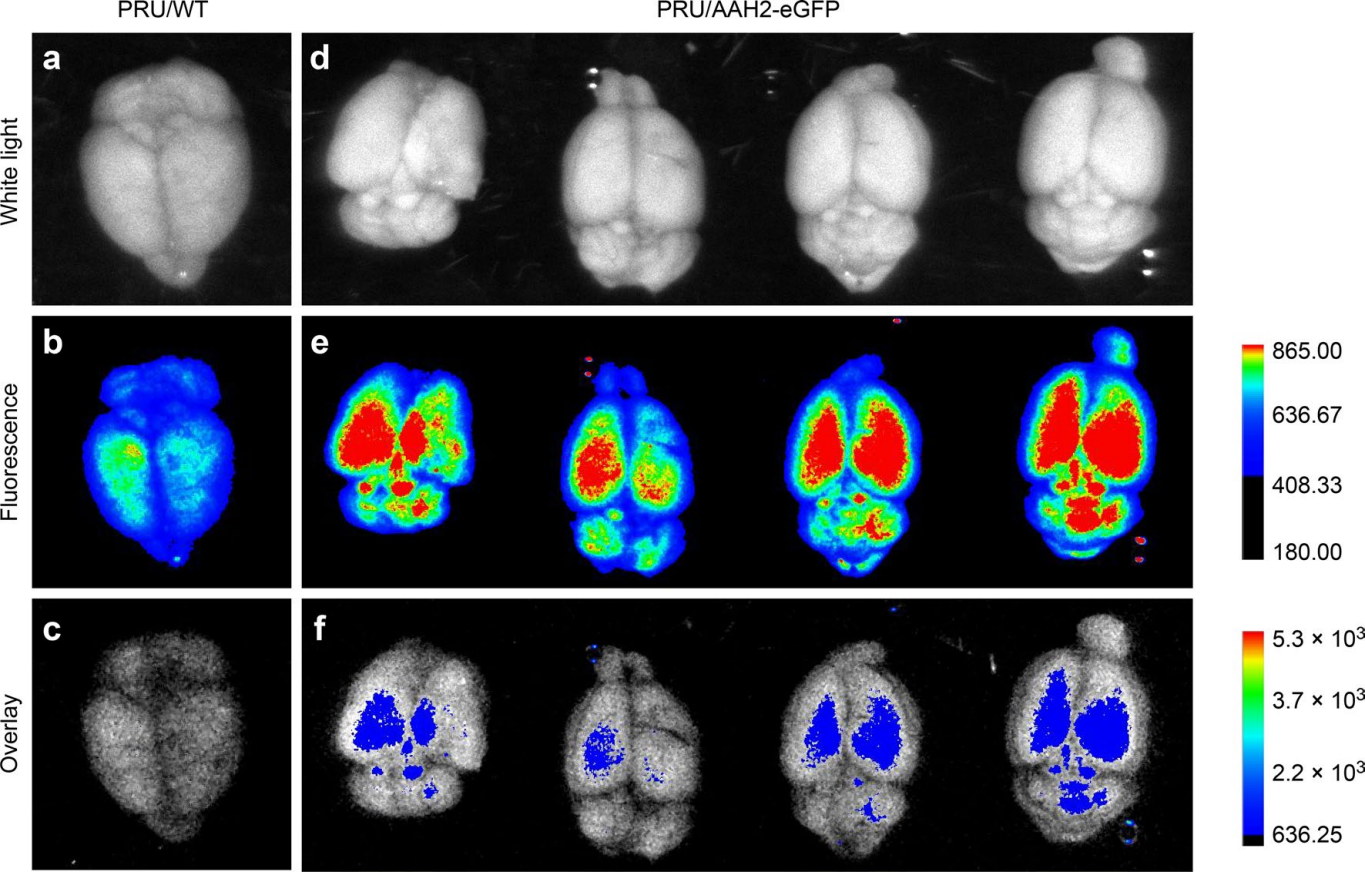
Images of brain tissue from the control mouse and chronically infected mice (Bruker *in Vivo* FX Pro). Morphological images of the brains were obtained with white light (**a**, **d**) and fluorescent images of the brains were obtained *via* the green fluorescence channels (**b**, **e**). Overlaid images of the brains were obtained by Bruker MI SE software (**c**, **f**)

To observe the fluorescence expression of the transgenic *T. gondii* cysts in the host, a chronic infection animal model of transgenic *T. gondii* was established by infecting Kunming mice with PRU/AAH2-eGFP. In contrast with the brains of PRU/WT tachyzoite-infected mice, the brains of the PRU/AAH2-eGFP-infected mice exhibited a bradyzoite-specific fluorescence-tagged AAH2 signal within cysts, showing that bradyzoites of transgenic *T. gondii* had formed *in vivo* and that a specific fluorescent signal for the transgenic *T. gondii* cysts could be observed (Fig. 4).

**Fig. 5:**
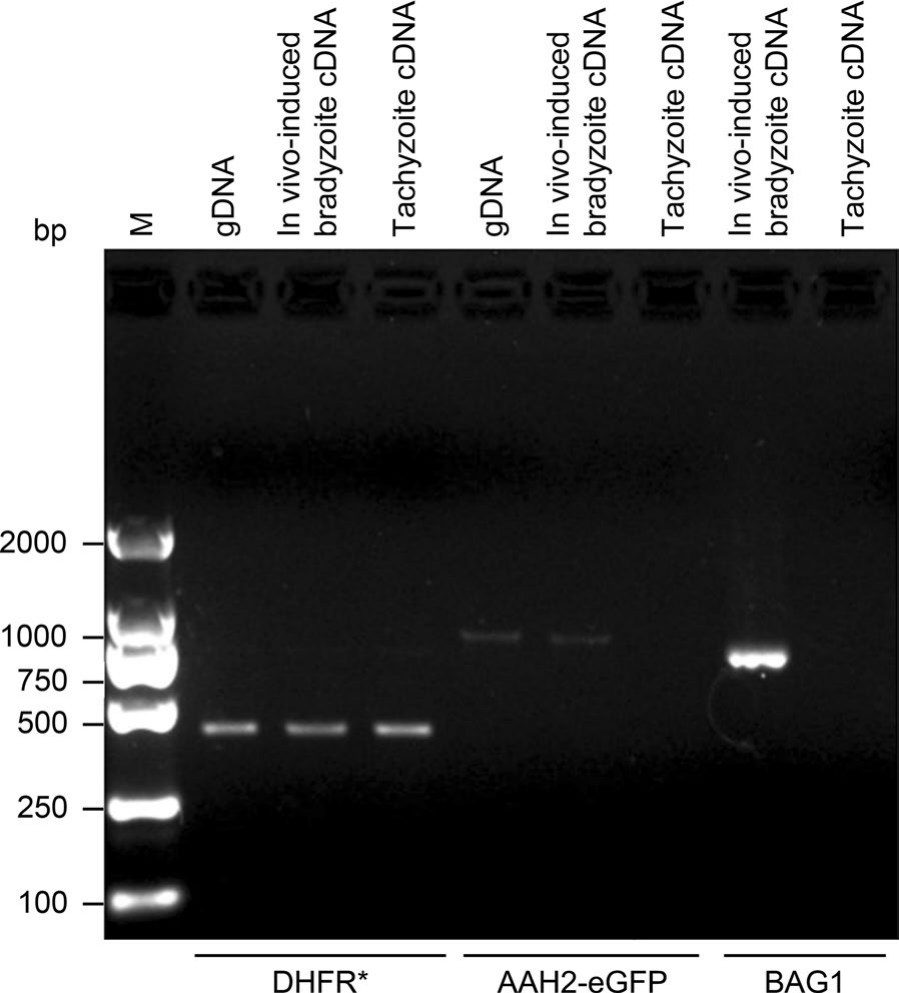
Identification of transgenic *T. gondii* cysts and expression of inserted genes in infected brain tissue. PCR verification of inserted DHFR* from different templates of PRU/AAH2-eGFP. PCR verification of AAH2-eGFP from different templates of PRU/AAH2-eGFP. RT-PCR verification of bradyzoite-specific BAG1 in PRU/AAH2-eGFP-infected brain tissue indicated the formation of cysts

RT-PCR was performed to further identify the status of the transgenic *T. gondii* PRU/AAH2-eGFP during the chronic phase. Amplification of the fragment of the BAG1 gene from infected brain tissue using primers P27 and P28 indicated that the *in vivo* induction of transgenic *T. gondii* bradyzoites was successful and that animal models of transgenic *T. gondii* chronic infection had been established. The positive fragments of the DHFR* gene were amplified by PCR from transgenic *T. gondii* gDNA, infected brain tissue cDNA and transgenic *T. gondii* tachyzoite cDNA using primers P33 and P34, and the results indicated that the resistance gene was stably expressed at both the tachyzoite and bradyzoite stages. Fragments of the AAH2-eGFP gene were only amplified *via* PCR using primers P35 and P36 from transgenic *T. gondii* gDNA and infected brain tissue cDNA and not from the cDNA of transgenic *T. gondii* tachyzoites, which showed that the bradyzoite-specific eGFP-tagged AAH2 gene had been transcribed and could be detected only at the bradyzoite stage as expected (Fig. 5).

The fluorescence images and verification PCR assay demonstrated that the transgenic *T. gondii* strain PRU/AAH2-eGFP was successfully generated by the TALEN technology and the inserted fluorescence gene and pyrimidine resistance gene were successfully expressed in this experiment.

## Discussion

### Amino acid hydroxylases from the bradyzoite stage of *T. gondii*

Acute infection with *T. gondii* can lead to teratogenesis, miscarriage and even death from toxoplasmic encephalitis in immunocompromised individuals; therefore, previous studies have mainly focused on the tachyzoite stage of *T. gondii* and the immune interaction between tachyzoite and the host, and the bradyzoite has been considered to have little effect on the host. Therefore, limited research has been performed on the interaction between bradyzoite-specific proteins and the host. However, in recent years, a growing number of studies have shown that *T. gondii* cysts caused by chronic infection (bradyzoite stage) are correlated with impairment of the function of the host brain and cognitive ability due to neurotropism. Therefore, our team focused on the study of bradyzoite-specific proteins and aimed to explore the interaction between the bradyzoites in cysts and neurotransmitters of the host.

Amino acid hydroxylases from *T. gondii* have received much attention in recent years in the context of interactions between parasites and hosts. Interestingly, the two amino acid hydroxylases in *T. gondii* mentioned previously share 98% sequence similarity with each other [16] and are homologous to phenylalanine hydroxylase. Attempts to delete AAH1 using the efficient CRISPR-Cas9 method have been unsuccessful to date [34] and AAH1 may be essential for the growth and development of parasites. Deletion of AAH2 did not affect tachyzoite or bradyzoite growth in tyrosine-rich or tyrosine-limiting medium [35], and the role of AAH2 in either stage of the life-cycle remains unidentified [13, 34]. Furthermore, recent studies have demonstrated that parasite-secreted AAH is not necessary for the generation of risky behaviour or the increased trappability observed during chronic *T. gondii* infection [36], and AAH2 is not required for tyrosine metabolism in these two developmental forms [35]. Recent studies have found that AAH2 is not required for the DA-dependent neurobehavioral abnormalities caused by *T. gondii* infection [37], but this subject remains controversial. Why is AAH2 expressed at a low level in tachyzoites and upregulated in bradyzoites [16]? This question is of great interest to researchers, which is why AAH2 was the first gene that we studied. In the course of this study, we established a method for editing targeted genes. Because the location of AAH2 in the brain is likely to be a critical factor for its effect on host behaviour, identifying this location during infection is crucial. Here, we generated an eGFP-tagged AAH2 *T. gondii* strain *via* TALEN technology to further characterize the localization of AAH2 in the bradyzoite stage.

### Genome editing technologies in *T. gondii*

In life science, research on gene functions is the basis for exploring pathogenic mechanisms, and advances in genome editing technologies have led to breakthroughs in the study of gene function. Artificial nucleases developed in recent years are tools that can conveniently and accurately edit genomes of interest. Currently, three main types of nucleases have been successfully used for genome editing: ZFNs, TALENs and CRISPR systems. CRISPR/CAS9 with a pyrimidine-resistant screening system is now widely used for genome editing of *T. gondii*, although the off-target effects of this system are caused by the recognition of target DNA by the single guide RNA (sgRNA) based on only 20 nt, and this drawback needs to be overcome. In contrast with the sgRNA of CRISPR/CAS9, the target DNA is recognized by repeat domains using TALEN technology. Each domain contains 33–35 amino acids, and one repeat domain corresponds to 1 nt, which guarantees the specificity of the cleavage site and leads to reduced off-target effects [32]. Here, we selected TALENs, a tool with reduced off-target effects, to develop an additional method to edit the *T. gondii* genome. In this study, the TALEN system was used to generate a transgenic *T. gondii* PRU strain, which was designated PRU/AAH2-eGFP. This strain could stably express an eGFP-tagged AAH2 fusion protein and was identified by verification PCR. The results demonstrate the feasibility of using TALEN technology for gene function research of *T. gondii* and provide a molecular technical basis for the mechanism of action of the *T. gondii* tyrosine hydroxylase in the host.

Although TALEN technology has high specificity and low off-target effects, it is complicated and time consuming to reconstruct TALEN plasmids to function normally in *T. gondii*. Using a *FastTALE* TALEN Assembly Kit, we achieved rapid assembly of TALEs, which recognize the target sequence. SAG1 is a 30-kDa surface protein of *T. gondii* tachyzoites that represents 3 to 5% of the total proteins in this parasite [33], and Soldati & Boothroyd [38] have successfully produced an RFP expression system with a SAG1 gene promoter. In this study, we recombined the TALEN plasmids using the SAG1 gene promoter to replace the CMV promoter for the expression of TALENs in *T. gondii* for gene editing. Transfection of recombinant plasmids containing the DHFR* mutation in individual bases into WT strains of *T. gondii* and the use of pyrimethamine to screen transgenic parasites have been widely used in transgenic technology [29, 30, 38, 39]. In the process of constructing the TALEN system, including the TALEN plasmids and donor plasmids as homologous templates, we also used a set of convenient methods for constructing recombinant plasmids, such as SOE PCR and In-Fusion technology.

### *In vitro* and *in vivo* studies of bradyzoite stage proteins

Due to the bradyzoite-specific expression of AAH2, the high-pH shock method was applied to induce tachyzoite-bradyzoite conversion of transgenic parasites *in vitro*. Upon successful bradyzoite induction of transgenic parasites, which was verified by RT-PCR of the bradyzoite-specific BAG1 gene, we observed that the reproduction of tachyzoites was distinctly retarded and that cyst-like structures could be observed under the conditions of the inducible medium. We hypothesized that the reproduction may have been changed by the conditions in the culture medium as well as by the conversion to the bradyzoite stage, which may have been induced simultaneously. However, no fluorescence was observed *in vitro*, and the transcriptional expression of AAH2-eGFP was not detected. The reasons underlying this observation may be as follows. First, expression of the BAG1 protein begins in the early stage of bradyzoites [40], and the AAH2 protein may not be expressed during short-term induction *in vitro*. Secondly, tachyzoite-bradyzoite conversion *in vitro* does not fully reflect the conditions of the cyst in the host and may affect the expression of the bradyzoite-specific gene.

To explore the condition of bradyzoites in the cyst in host, chronic infection models were established in this study to further analyse the expression of the AAH2 protein in the host. In contrast with those of the control mice, fluorescence images and verification PCR of mice infected by transgenic parasites demonstrated that the inserted fluorescence gene and pyrimidine resistance gene were successfully expressed. The successful construction of an AAH2 fluorescent marker strain PRU/AAH2-eGFP allowed us to further study the secretion process of AAH2 protein. Studies have found that AAH2 has a signal sequence motif, suggesting that the protein is a secreted protein. Using PRU/AAH2-eGFP, we can further explore whether the AAH2 protein can be secreted outside the cysts during the bradyzoite stage and interact with the host brain tissue, which will provide useful clues for further study of the regulatory role of tyrosine hydroxylase of *T. gondii* in the host dopamine metabolic pathway. Researchers are interested in tachyzoite-bradyzoite interconversion and have established an *in vitro* model of parasite interconversion in host cells; however, few *in vivo* studies have been performed on the interaction of bradyzoite-specific proteins with the host nervous system. The results of this study indicate that studies of bradyzoite-stage proteins are best conducted in host models *in vivo* because the interconversion of parasites in the host is closely associated with host immunity. The present study provides a convenient and quick method to examine the fluorescence localization of bradyzoite-specific proteins, namely *in vivo* imaging. *In vivo* research is the ultimate tool for the investigation of biological reactions, and direct imaging results can provide clear information at a glance.

## Conclusions

The generation of PRU/eGFP-AAH2 indicates that TALENs can specifically target and successfully edit specific genes in the *T. gondii* genome, thus providing a strategy for exploring *T. gondii* gene function. However, certain limitations were observed in our study, and further research is required for the construction of fluorescence-tagged AAH2 to be used to trace the secretory pathway of AAH2 in parasites and further study the interactions of the parasite with the host nervous system. A transgenic *T. gondii* that can stably express fused bradyzoite stage-specific fluorescent protein can be used to determine the localization of AAH2, which may indicate the role of the parasite’s AAH2 in host-parasite interactions and could contribute to further understanding the upregulation of AAH2 in bradyzoites.

## Methods

### Mice

Female 6-week-old Kunming mice were purchased from the Animal Experimental Centre of Southern Medical University (Guangzhou, China). The Kunming mice were infected with parasites, and their brain tissue was excised after euthanasia by cervical dislocation. Fluorescence localization of the AAH2 protein in the brain tissue was evaluated using an *in vivo* imaging system (Bruker FX Pro, Billerica, MA. USA).

### Cells and parasite cultures

The PRU strain of *T. gondii* was maintained in human foreskin fibroblasts (HFFs) cultured in tachyzoite medium [Dulbecco’s modified Eagle’s medium (DMEM; Gibco, Beijing, China), 1% foetal bovine serum (FBS; Gibco, Newcastle, Australia), 1% penicillin and streptomycin (Gibco, Frederick, MD.USA)] at 37 °C in a 5% CO_2_ incubator. All PRU strain parasites were maintained until the parasites lysed 80–100% of the host cells. For the collection of tachyzoites, the parasites were syringed with a 27-gauge needle, filtered through a 3.0-μm-pore filter and then pelleted at 2500× *rpm* for 10 min.

### Construction of TALEN plasmids and homology template

The primer sequences and plasmid structures used in this study are listed in the additional files (Additional file 3: Table S1 and Additional file 4: Table S2). The sequences for primer design and targeting plasmid validation were obtained from ToxoDB (www.toxodb.org) [41].

The TALENs were constructed with the *FastTALE* TALEN Assembly Kit (Sidansai Biotechnology Co., Shanghai, China). The steps were as follows. According to the sequencing result for the *T. gondii* PRU strain tyrosine hydroxylase type2 (AAH2, locus on chromosome V, annotated as TGME49_212740), we first selected a pair of suitable DNA sequences for gene targeting and named the pair TALE-L/R (Additional file 5: Figure S3). We assembled TALEN modules that recognized one or two bases (Sidansai Biotechnology Co.) into a suitable skeleton vector (pL62 for L, pR54 for R) that contained CMV promoter and *Fork* I sequence and constructed pTALEN-L and pTALEN-R using a *FastTALE* TALEN Assembly Kit according to the manufacturer’s protocol (Additional file 6: Figure S4a, b).

To recombine pTALEN-L, the SAG1 gene promoter region (SAG1p-L) was amplified by PCR from genomic DNA (gDNA) isolated from *T. gondii* PRU strain tachyzoites (DNeasy Blood & Tissue Kit; Qiagen, Hilden, Germany) cultured *in vitro* as previously described [42] using primers P1 and P2. The terminal region of the GRA2 gene (GRA2t-L) was amplified by PCR from gDNA using primers P3 and P4. The amplified SAG1p-L fragment was digested with the same enzymes used to digest pTALEN-L, namely *Asc*I and *Spe*I-HF, and the resulting plasmid was designated pTALEN-L-SAG1. The amplified GRA2t-L fragment was digested with the same enzymes used to digest pTALEN-L-SAG1, namely *Bgl*II and *Not*I-HF, and the resulting plasmid was designated pTALEN-L-SAG1-GRA2 (pTALEN-L-SG) (Additional file 6: Figure S4c).

To recombine pTALEN-R, the SAG1 gene promoter region (SAG1p-R) was amplified by PCR from gDNA using primers P5 and P6. The terminal region of the GRA2 gene (GRA2t-R) was amplified by PCR from gDNA using primers P7 and P8. In addition, the pyrimethamine-resistant DHFR (DHFR*) was amplified from the pLic3×HA-DHFR-TS plasmid using primers P9 and P10. The amplified GRA2t-R fragment was digested with the same enzymes used to digest pTALEN-L-GRA2, namely *Bgl*II and *Not*I-HF, and the resulting plasmid was designated pTALEN-R-GRA2. The amplified SAG1p-R fragment and DHFR* were digested with the same enzymes used to digest pTALEN-R-GRA2, namely *Asc*I and *Spe*I, and the resulting plasmid was designated pTALEN-R-DHFR-TS-SAG1-GRA2 (pTALEN-R-DSG) (Additional file 6: Figure S4d).

To construct a donor plasmid as a homology template, the left arm (Z) was amplified from PRU strain gDNA as a homologous fragment using primers P11 and P12. The right arms (Y1, Y2) of the AAH2 gene were amplified from PRU strain gDNA as homologous fragments using primers P13/P14 and P15/P16. The eGFP gene (E) was amplified from the pR52 plasmid using primers P17 and P18. DHFR* (D) was amplified from pLic3×HA-DHFR-TS using primers P19 and P20. These amplified fragments Z, E, D, Y1, and Y2 were extended with the spliced-overlapping-extension (SOE) method and then amplified by PCR (the SOE PCR details are described in Additional file 7: Table S3). The SOE PCR product ZEDY was inserted into the plasmid pUC19 (TaKaRa, Dalian, China), which was linearized by digestion with *BamH*I using the In-Fusion enzyme (Clontech, Mountain View, CA. USA), and the resulting donor plasmid was designated pZEDY (Additional file 8: Figure S5).

### Generation of the transgenic *T. gondii* PRU strain

To generate transgenic parasites expressing eGFP-tagged AAH2 during the bradyzoite stage, electroporation of tachyzoites was performed as previously described [43]. *Toxoplasma gondii* PRU strain tachyzoites were purified [42] and 4 × 10^7^ tachyzoites were resuspended with 24 μg of donor plasmid and approximately 63 μg of TALEN plasmid (21 μg of pTALEN-L-SG and 42 μg of pTALEN-R-DSG) in 400 μl of cytomix buffer [120 mM KCl, 0.15 mM CaCl_2_, 10 mM K_2_HPO_4_-KH_2_PO_4_, 25 mM HEPES (pH 7.6), 2 mM EDTA (pH 7.6), 5 mM MgCl_2_, 2 mM ATP and 5 mM GSH (pH 7.6)] [44]. The parasite suspension was transferred to 0.4-cm-gap cuvettes (Bio-Rad Laboratories, Hercules, CA, USA), and electroporation was performed at 1500 V, 25 Ω, and 25 μF using a Gene Pulser Xcell™ electroporation system (Bio-Rad Laboratories). After electroporation, the parasites were incubated for 20 min at room temperature. Then, the parasites were inoculated into HFFs in 25-cm^2^ T-flasks (Corning, Wujiang, China) and cultured in a conventional environment with drug-free culture medium as mentioned above for 24 h to allow for the action of TALENs on the target gene and the expression of the pyrimethamine-resistant DHFR. Then, pyrimethamine (1 μM; Sigma-Aldrich, Buchs, Switzerland) was added to the medium. Stable transgenic parasites were selected in DMEM supplemented with high concentrations of pyrimethamine (2–3 μM) for 20 generations (2nd to 15th generation, 2 μM; 16th and above, 3 μM) [45]. After screening of several generations, the pyrimethamine-resistant parasites were identified by PCR. The confirmed parasites were designated PRU/AAH2-eGFP, and they could express eGFP-tagged AAH2 during the bradyzoite stage (cyst stage).

### Tachyzoite-bradyzoite conversion of PRU/AAH2-eGFP *in vitro*

The high-pH shock method [46] was applied to induce the stage conversion of tachyzoites to bradyzoites *in vitro*. First, 5 × 10^4^ PRU/AAH2-eGFP tachyzoites were inoculated into HFFs in 25-cm^2^ T-flasks and cultured for 4 h in tachyzoite medium to allow tachyzoites to invade the cells. To establish a tachyzoite-to-bradyzoite interconversion system, the tachyzoite medium was replaced with bradyzoite medium [RPMI 1640 (Gibco, Beijing, China), 1 g/500 ml NaHCO_3_ (Sigma-Aldrich, Saint. Louis, MO. USA), 10 mM HEPES (Sigma-Aldrich, Saint. Louis, MO. USA), 1% FBS and 1% penicillin and streptomycin at pH 8.2] as a test group, and the lid of the flask was sealed to isolate parasites from the 5% CO_2_ environment to maintain an alkaline pH. Additionally, the medium was changed daily. Simultaneously, the tachyzoite medium in another flask was replaced with tachyzoite medium as a control. Parasite morphology was observed using an inverted microscope (Nikon, Tokyo, Japan), and the expression of fluorescence was observed daily by inverted fluorescence microscopy (Nikon). After 5 days of induction, two groups of parasites were purified to extract RNA and establish cDNA for identification.

### Tachyzoite-bradyzoite conversion of PRU/AAH2-eGFP *in vivo*

To establish the chronically infected model, 6-week-old female Kunming mice (*n* = 4) were infected intraperitoneally (i.p.) with PRU/AAH2-eGFP, and one mouse was i.p. infected with PRU/wild-type (WT) tachyzoites as a control. Because the skulls and hair of mice would interfere with imaging, the brains of the PRU/AAH2-eGFP-infected mice and PRU/WT-infected mouse were excised to obtain images of fluorescence expression in bradyzoites induced *in vivo* after 3 months of infection. The brain tissue was kept intact and washed in normal saline to remove blood and stained hair and then placed in a disposable culture dish for photography. Standard exposure and an optimized exposure time were used. A multiwavelength illumination source and an excitation wavelength of 498 nm and emission wavelength of 516 nm were selected according to the fluorophore of the sample (eGFP). After the programme was established, the brain samples were directly imaged using a Bruker *in vivo* imaging system (Bruker FX Pro) to observe the fluorescence localization of the AAH2 protein during the latent phase (cyst stage) *in vivo*. Then, the brain tissues were immediately ground into homogenate for RT-PCR identification.

## Additional files

**Additional file 1: Figure S1.** Construction and identification of TALEN plasmids.

**Additional file 2: Figure S2.** Identification of the recombinant donor plasmid pZEDY.

**Additional file 3: Table S1.** Nucleotide sequences of all primers designed in this study.

**Additional file 4: Table S2.** Structures of the plasmids used in this study.

**Additional file 5: Figure S3.** TAL effector nucleases designed for specific targeting of TgAAH2.

**Additional file 6: Figure S4.** Steps for constructing TALEN plasmids.

**Additional file 7: Table S3.** SOE PCR to extend and amplify ZEDY.

**Additional file 8: Figure S5.** Schematic of donor plasmid construction.

## Abbreviations

AAH2: aromatic amino acid hydroxylase 2
eGFP: enhanced green fluorescence protein
TH: tyrosine hydroxylase
AaaH: aromatic amino acid hydroxylase
HR: homologous recombination
ZFNs: zinc finger nucleases
TALENs: transcription activator-like effector nucleases
CRISPR/Cas: clustered regularly interspaced palindromic repeats/CRISPR-associated proteins
TALEs: transcription activator-like effector nucleases
DSB: double-strand break
HFFs: human foreskin fibroblasts
FBS: foetal bovine serum
SOE: spliced-overlapping-extension
WT: wild type
