# Supplementary material for: Efficient genome engineering of Toxoplasma gondii using the TALEN technique
Source: Parasit Vectors. 2019 Mar 15;12:112. doi: 10.1186/s13071-019-3378-y (PMC6419828; doi:10.1186/s13071-019-3378-y)
Supplement: Supplementary file 1 — Additional file 1: Figure S1. Construction and identification of TALEN plasmids. [file 13071_2019_3378_MOESM1_ESM.docx]

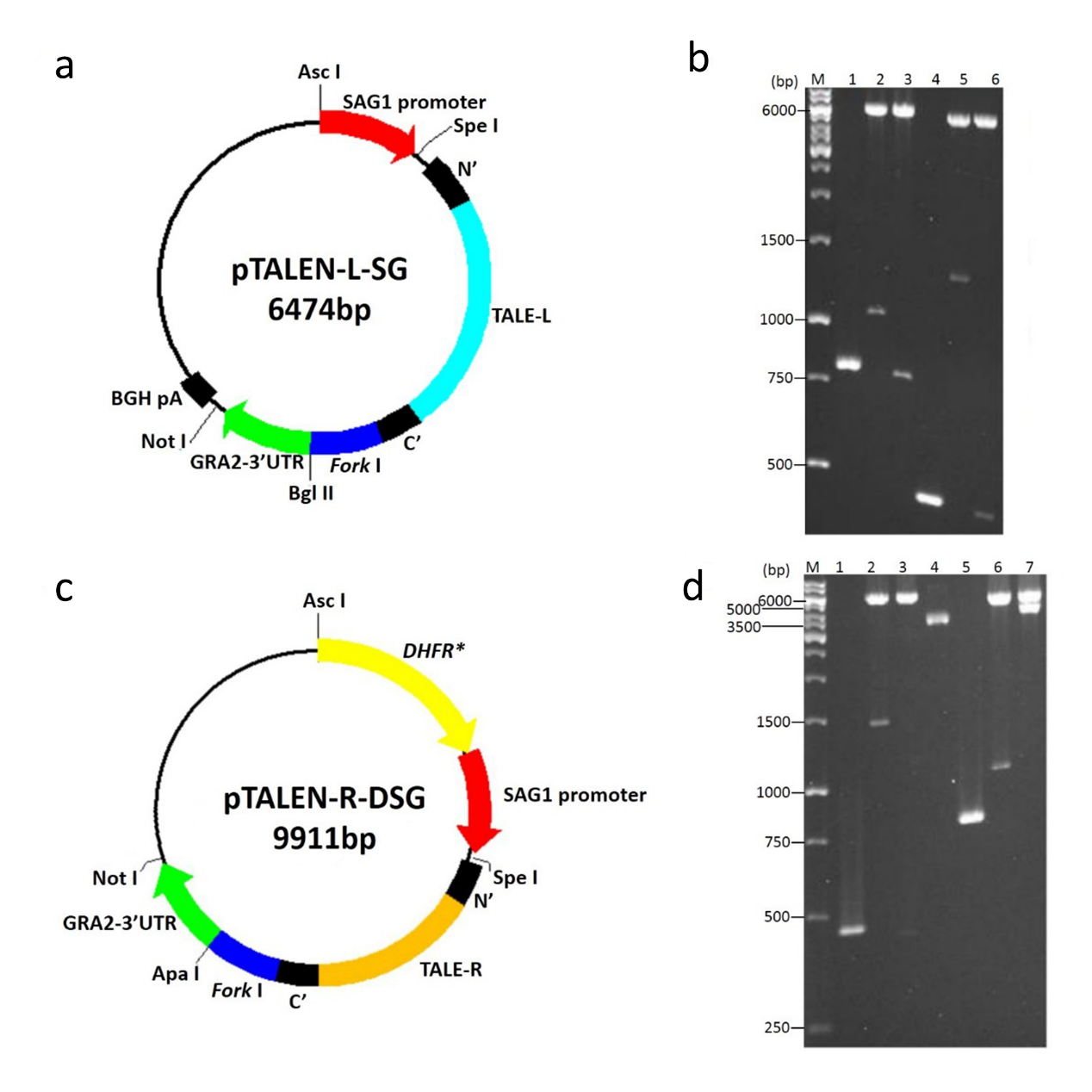


**Figure S1. Construction and identification of TALEN plasmids. a** Schematic of the recombinant plasmid pTALEN-L-SG. **b** Identification electrophoresis map of pTALEN-L-SG. M: GeneRuler 1 kb DNA ladder, 1: amplified fragment of the SAG1 promoter, 2: pTALEN-L digested with *Asc*I/ *Spe*I, 3: SAG1 promoter from the recombinant plasmid pTALEN-L-SAG1 after digestion with *Asc*I/*Spe*I, 4: amplified fragment of the GRA2 terminator, 5: pTALEN-L-SAG1 digested with *Bgl*II/*Not*I, 6: GRA2 terminator from the recombinant plasmid pTALEN-L-SG after digestion with *Bgl*II/*Not*I. **c** Schematic of the recombinant plasmid pTALEN-R-DSG. **d** Identification electrophoresis map of pTALEN-R-DSG. M: GeneRuler 1 kb DNA ladder, 1: amplified fragment of the GRA2 terminator, 2: pTALEN-R digested with *Apa*I/*Not*I, 3: GRA2 terminator from the recombinant plasmid pTALEN-R1-GRA2 after digestion with *Apa*I/*Not*I, 4: the whole sequence of DHFR*, 5: amplified fragment of the SAG1 promoter, 6: pTALEN-R-GRA2 digested with *Asc*I/*Spe*I, 7: The DHFR* fragment and SAG1 promoter from pTALEN-R-DSG after digestion with *Asc*I/*Spe*I.
