# Supplementary material for: Efficient genome engineering of Toxoplasma gondii using the TALEN technique
Source: Parasit Vectors. 2019 Mar 15;12:112. doi: 10.1186/s13071-019-3378-y (PMC6419828; doi:10.1186/s13071-019-3378-y)
Supplement: Supplementary file 2 — Additional file 2: Figure S2. Identification of the recombinant donor plasmid pZEDY. [file 13071_2019_3378_MOESM2_ESM.docx]

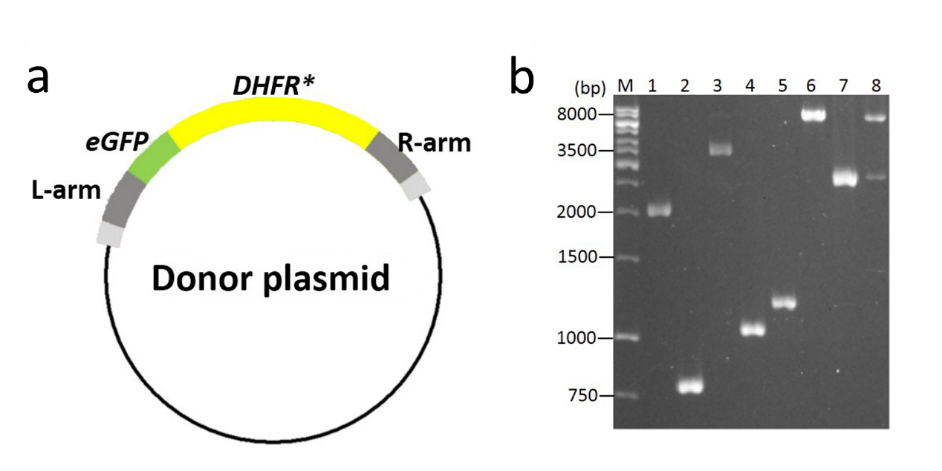


**Figure S2. Identification of the recombinant donor plasmid** **pZEDY. a** Schematic of the donor plasmid pZEDY. **b** Identification electrophoresis map of pZEDY. M: 1 kb DNA ladder, 1: Z - homologous left arm, 2: E - eGFP gene, 3: D - DHFR*, 4: Y1 - the first half of the homologous right arm, 5: Y2 - the second half of the homologous right arm, 6: the SOE PCR product ZEDY, 7: the PCR product of pUC19 containing 2 BamHI sites, 8: pZEDY digested with BamHI.
