# Supplementary material for: Efficient genome engineering of Toxoplasma gondii using the TALEN technique
Source: Parasit Vectors. 2019 Mar 15;12:112. doi: 10.1186/s13071-019-3378-y (PMC6419828; doi:10.1186/s13071-019-3378-y)
Supplement: Supplementary file 3 — Additional file 3: Table S1. Nucleotide sequences of all primers designed in this study. [file 13071_2019_3378_MOESM3_ESM.docx]

**Table S1.** Nucleotide sequences of all primers designed in this study.

| Gene or locus | Primer Sequence [5’ 🡪 3’] | Restriction enzyme |
| --- | --- | --- |
| Amplification of TALEN plasmids | | |
| SAG1p-L (P1) | CAGATATACGGCGCGCCTTACATCCGTTGCCTTTTCCAC | AscI |
| SAG1p-L (P2) | ACCACACTGGACTAGTCTCGCCAAAAAACCAGAAGAA | SpeI |
| GRA2t-L (P3) | GATCAACTTCAGATCT**TGATAA**GACTACGACGAAAGTGATGCG | BglII |
| GRA2t-L (P4) | AACGGGCCCGCGGCCGCGTCGACTGGAACATCGGTGT | NotI |
| SAG1p-R (P5) | TGCACTTGCAGGATGCGAAGCTTTTACATCCGTTGCCT |  |
| SAG1p-R (P6) | ACCACACTGGACTAGTGGAAACATACTCGCCAAAAAACCAG | SpeI |
| GRA2t-R (P7) | TTTTGGCAATGTGAGGGCCCGACTACGACGAAAGTGATGCG | ApaI |
| GRA2t-R (P8) | CAGTAGAAGGCGGCCGCGTCGACTGGAACATCGGTGT | NotI) |
| DHFR*-TS-R (P9) | CAGATATACGGCGCGCCAAGCTTCGCCAGGCTGTAA | AscI |
| DHFR*-TS-R (P10) | CATCCTGCAAGTGCATAGAAG |  |
| Amplification of homologous arms | | |
| AAH2 left arm, Z (P11) | CGGTACCCGGGGATCCGTCCACATTGGTCGTGAAAC | BamHI |
| AAH2 left arm, Z (P12) | **CCCTTGCTCACCAT**GATCTTGAGGGAGACAGGAGG |  |
| AAH2 right arm 1, Y1 (P13) | **GTATGCTATACGAAGTTAT**ACCAGACACTGTATCTCTACTTTGC |  |
| AAH2 right arm 1, Y1 (P14) | **CTTCTTCGTGGTTTCCCTCA** |  |
| AAH2 right arm 2, Y2 (P15) | **GGGAAACCACGAAGAAGCC** |  |
| AAH2 right arm 2, Y2 (P16) | CGACTCTAGAGGATCCCTGGAACGAGGGTAGGAGGT | BamHI |
| eGFP (P17) | **CCTCCTGTCTCCCTCAAGATC**ATGGTGAGCAAGGG |  |
| eGFP (P18) | **TTACAGCCTGGCGAAGCTT**ATAACTTCGTATAGCATACATTATACGAAGTTATTTACTTGTACAGCTCGTC | Loxp |
| DHFR* (P19) | **TGTATGCTATACGAAGTTAT**AAGCTTCGCCAGGCTGTAA |  |
| DHFR* (P20) | **GTAGAGATACAGTGTCTGGT**ATAACTTCGTATAGCATACATTATACGAAGTTATCATCCTGCAAGTGCATAGAAG | Loxp |
| pUC19 (P21) | GGATCCTCTAGAGTCGACCTGC | BamH1 |
| pUC19 (P22) | GGATCCCCGGGTACCGAGCTC | BamH1 |
| PCR1-F (P23) | CATTGGCTCTTTCGAGATGTCG |  |
| PCR1-R (P24) | TGAACTTCAGGGTCAGCTTGC |  |
| PCR2-F (P25) | AGGGATGACTCTTCATGTGGC |  |
| PCR2-R (P26) | ACTGAGGGGATCGTCATATCGG |  |
| BAG1-F (P27) | ACATGAATTCATGGCGCCGTCAGCATCGCA |  |
| BAG1-R (P28) | CGACAAGCTTCTACTTCACGCTGATTTGT |  |
| SAG1-F (P29) | GTCTGAATTCATGTCGGTTTCGCTG |  |
| SAG1-R (P30) | AATTCTCGAGTCACGCGACACAAGC |  |
| eGFP-F (P31) | ATGCGTGCTGGCGTCTTCTGCT |  |
| eGFP-R (P32) | CGATGCCAAGGGCGTTATCTGGT |  |
| DHFR*-F (P33) | GACCCCTCGTGGACAGATTG |  |
| DHFR*-R (P34) | GATGGGTCGATACGTCGCTT |  |
| AAH2-eGFP (P35) | CTCGAGGTTCGTCAGTGG |  |
| AAH2-eGFP (P36) | CGTCCATGCCGAGAGTGAT |  |
