# Supplementary material for: Efficient genome engineering of Toxoplasma gondii using the TALEN technique
Source: Parasit Vectors. 2019 Mar 15;12:112. doi: 10.1186/s13071-019-3378-y (PMC6419828; doi:10.1186/s13071-019-3378-y)
Supplement: Supplementary file 4 — Additional file 4: Table S2. Structures of the plasmids used in this study. [file 13071_2019_3378_MOESM4_ESM.docx]

**Table S2.** Structures of the plasmids used in this study.

| Label | Structure |
| --- | --- |
| Skeleton Vector L62 | sCMV-SP6-3xFlag-NLS-N’-[TALE]-1/2C-C’-FokI-IRES-RFP-pA |
| Skeleton Vector R52 | sCMV-SP6-3xFlag-NLS-N’-[TALE]-1/2T-C’-FokI-IRES-EGFP-pA |
| pTALEN-L-SG | SAG1p-L-SP6-3xFlag-NLS-N’-TALE-L-1/2T-C’-FokI-GRA2t-L |
| pTALEN-R-DSG | DHFR-TS-SAG1p-R-SP6-3xFlag-NLS-N’-TALE-R-1/2T-C’-FokI- GRA2t-R |
| pLic3×HA-DHFR-TS | 3×HA-DHFR-TS-f1ori-AMP-PBR322 ori |
| pZEDY | pUC19BamHI upstream fragment-left homologous arm-eGFP-Loxp-DHFR*-Loxp-right homologous arm- pUC19BamHI downstream fragment |
