# Supplementary material for: Efficient genome engineering of Toxoplasma gondii using the TALEN technique
Source: Parasit Vectors. 2019 Mar 15;12:112. doi: 10.1186/s13071-019-3378-y (PMC6419828; doi:10.1186/s13071-019-3378-y)
Supplement: Supplementary file 5 — Additional file 5: Figure S3. TAL effector nucleases designed for specific targeting of TgAAH2. [file 13071_2019_3378_MOESM5_ESM.docx]

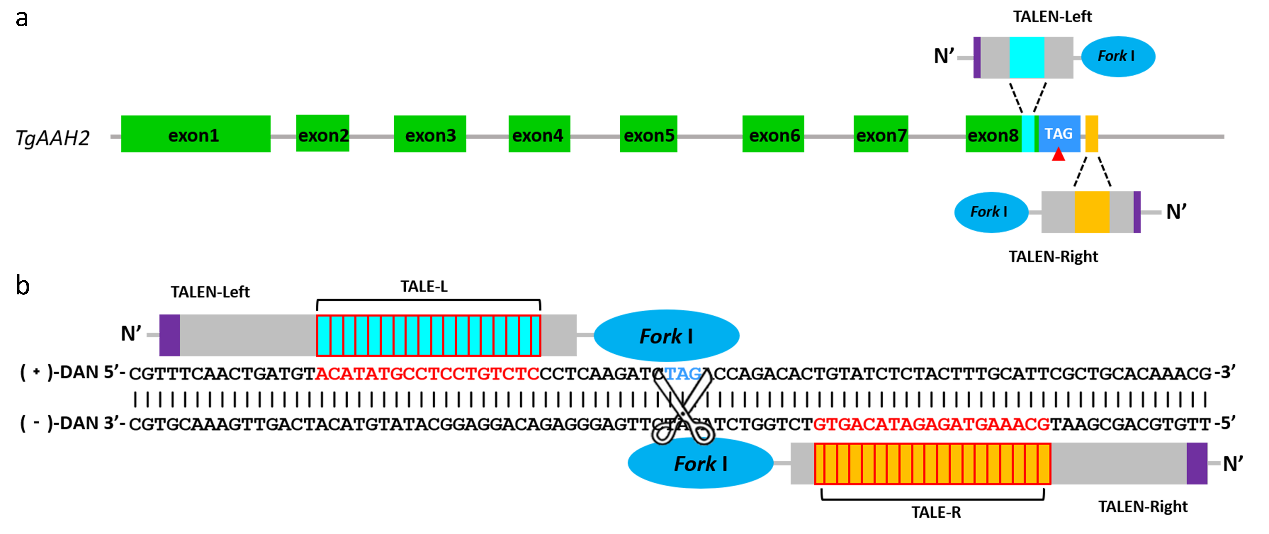


Figure S3. **TAL effector nucleases designed for specific targeting of** **TgAAH2.** **a** Schematic representation of AAH2 in the *T. gondii* PRU strain genome and the target sites of the TALENs. A TALEN is composed of three important functional regions: a nuclear localization signal (NLS) at the N terminus (purple), a central repeat domain (bluish green/orange) that identifies DNA sites, and a *Fork* I catalytic domain at the C terminus (blue) [32]. Two TALENs identify and execute cleavage near the stop codon (TAG). **b** Schematic of a pair of TALENs (TALEN-L/R) binding to two neighbouring target sequences (red) of TgAAH2, allowing *Fork* I domains to dimerize and cleave DNA.
