# Supplementary material for: Efficient genome engineering of Toxoplasma gondii using the TALEN technique
Source: Parasit Vectors. 2019 Mar 15;12:112. doi: 10.1186/s13071-019-3378-y (PMC6419828; doi:10.1186/s13071-019-3378-y)
Supplement: Supplementary file 6 — Additional file 6: Figure S4. Steps for constructing TALEN plasmids. [file 13071_2019_3378_MOESM6_ESM.docx]

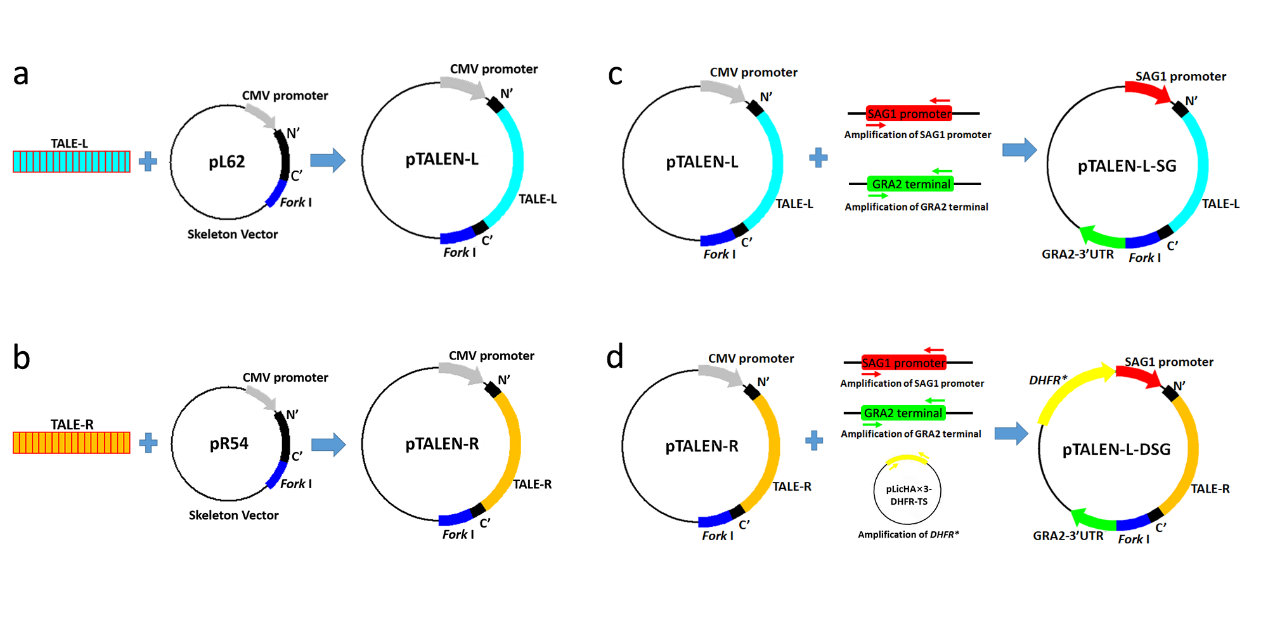


Figure S4. **Steps for constructing TALEN plasmids. a** Schematic of the skeleton vector reconstructed into pTALEN-L using the *FastTALE* TALEN Assembly Kit. **b** Schematic of the skeleton vector reconstructed into pTALEN-R using the *FastTALE* TALEN Assembly Kit. **c** Schematic of pTALEN-L recombination into pTALEN-L-SG by PCR and double digestion. **d** Schematic of pTALEN-R recombination into pTALEN-R-DSG by PCR and double digestion.
