# Supplementary material for: Efficient genome engineering of Toxoplasma gondii using the TALEN technique
Source: Parasit Vectors. 2019 Mar 15;12:112. doi: 10.1186/s13071-019-3378-y (PMC6419828; doi:10.1186/s13071-019-3378-y)
Supplement: Supplementary file 7 — Additional file 7: Table S3. SOE PCR to extend and amplify ZEDY. [file 13071_2019_3378_MOESM7_ESM.docx]

**Table S3.** SOE PCR to extend and amplify ZEDY.

| First reaction in the PCR instrument. the PCR parameters were as follows: 98°C for 4 min; 20 cycles of 98°C for 10 s, 62-68°C for 1 min, and 68°C for 5 min 36 s; and 68°C for 7 min. The reaction system was as follows:   \| Reaction System \| \| \| --- \| --- \| \| RNase-free water \| 31.4 μl \| \| 5×PrimeSTAR GXL buffer \| 10 μl \| \| dNTPs (2.5 mM) \| 4 μl \| \| Z (79.3 ng/μl) \| 0.5 μl \| \| E (43.8 ng/μl) \| 1 μl \| \| D (137 ng/μl) \| 0.3 μl \| \| Y1 (80.3 ng/μl) \| 0.5 μl \| \| Y2 (138 ng/μl) \| 0.3 μl \| \| PrimeSTAR GXL DNA polymerase (1.25U) \| 1 μl \| \| Total volume \| 49 μl \| |
| --- | --- | --- | --- | --- | --- | --- | --- | --- | --- | --- | --- | --- | --- | --- | --- | --- | --- | --- | --- | --- | --- | --- |
| Second reaction in the PCR instrument. The PCR tubes were placed on ice, and 0.5 μl of the primers Z-F (20 μM) and Y2-R (20 μM) were added into the reactions. Then, the PCR tubes were placed back into the PCR machine, and the following parameters were used: 98°C for 4 min; 30 cycles of 98°C for 10 s, 62-68°C for 15 s, 68°C for 8 min 31 s; and 68°C for 7 min. |
