# Supplementary material for: Efficient genome engineering of Toxoplasma gondii using the TALEN technique
Source: Parasit Vectors. 2019 Mar 15;12:112. doi: 10.1186/s13071-019-3378-y (PMC6419828; doi:10.1186/s13071-019-3378-y)
Supplement: Supplementary file 8 — Additional file 8: Figure S5. Schematic of donor plasmid construction. [file 13071_2019_3378_MOESM8_ESM.docx]

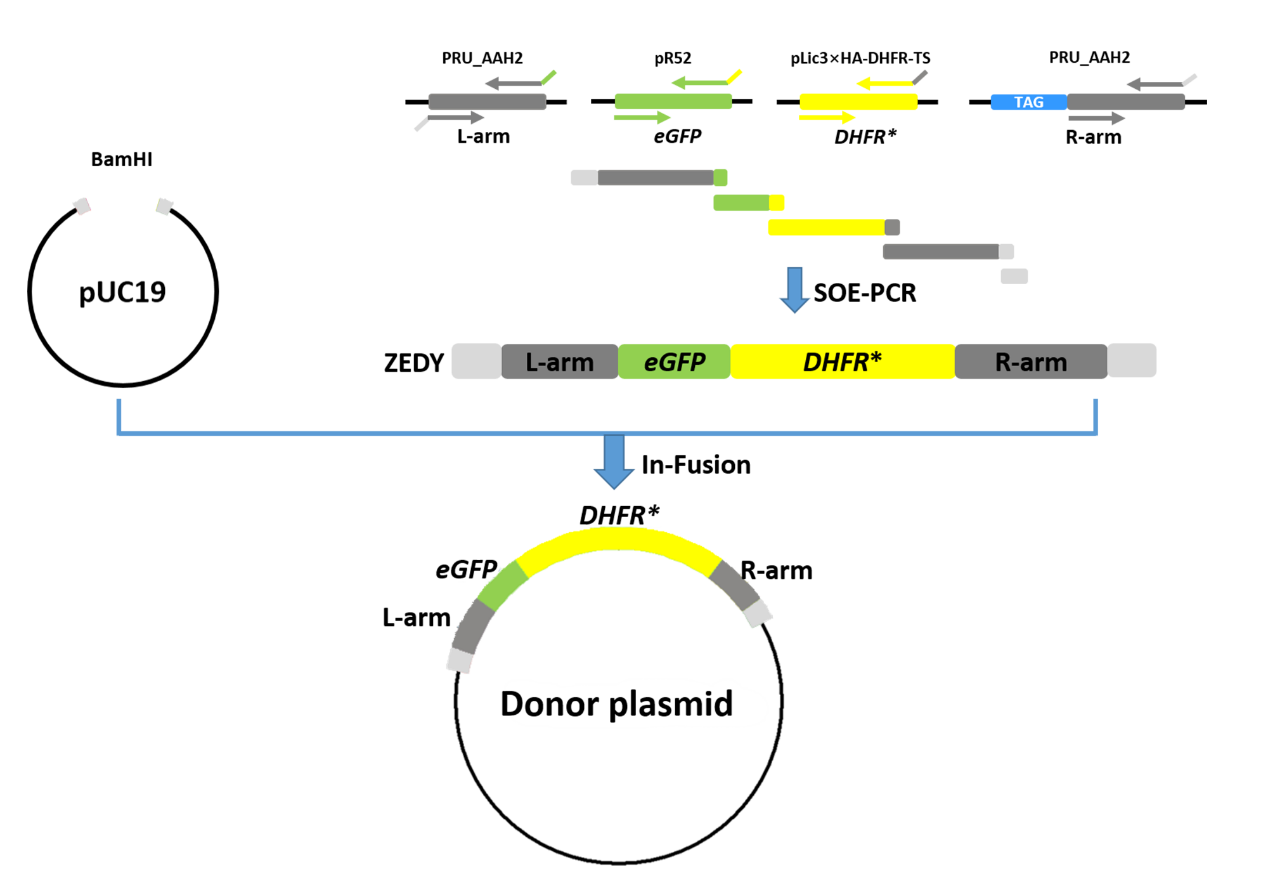


Figure S5. **Schematic of donor plasmid construction.** Schematic of donor plasmid construction by recombination of the homology template and pUC19 vector by SOE PCR, restriction enzyme digestion and In-Fusion technique.
